# Determining Static Hyperinflation in Patients with Severe Emphysema: Relation Between Lung Function Parameters and Patient-Related Outcomes

**DOI:** 10.1007/s00408-020-00368-9

**Published:** 2020-06-28

**Authors:** Wouter W. de Weger, Karin Klooster, Nick H. ten Hacken, Marlies van Dijk, Jorine E. Hartman, Dirk-Jan Slebos

**Affiliations:** Department of Pulmonary Diseases AA11, University of Groningen, University Medical Center Groningen, Hanzeplein 1, 9713 GZ Groningen, The Netherlands

**Keywords:** Hyperinflation, Emphysema, Residual volume, Lung volume reduction, COPD

## Abstract

**Background:**

Bronchoscopic lung volume reduction techniques are minor invasive treatment modalities for severely hyperinflated emphysema patients. The severity of static lung hyperinflation determines eligibility and success rate for these treatments. However, it is not exactly known what parameter should be used to optimally reflect hyperinflation. Commonly used parameters are residual volume (RV) and the RV/Total lung capacity (TLC) ratio. Other parameters reflecting hyperinflation are Inspiratory Capacity/TLC and forced vital capacity.

**Objectives:**

To define which of these function parameters is the most optimal reflection of hyperinflationin in relation to patient-related outcomes.

**Methods:**

In a retrospective cohort study, data from measurements during baseline visits of eight studies were pooled. Primary outcomes were RV/TLC ratio and RV as percentage of predicted (RV%pred), both measured by bodyplethysmography, compared to the patient-related outcome variables: 6-min walk distance (6MWD), the St. George’s Respiratory Questionnaire (SGRQ), and the modified Medical Research Council (mMRC).

**Results:**

Two hundred seventy-four COPD patients (mean age 59 years; 66% female), FEV_1_ 0.74 ± 0.28 L, RV 4.94 ± 1.06 L, 6MWD of 339 ± 95 m, were included in the analysis. Significant correlations (all *p* < 0.01) were found between RV%pred and 6MWD (*r* =  − 0.358), SGRQ (*r* = 0.184), and mMRC (*r* = 0.228). Also, there was a significant correlation between RV/TLC ratio and 6MWD (*r* =  − 0.563), SGRQ (*r* = 0.289) and mMRC (*r* = 0.354). Linear regression analyses showed that RV/TLC ratio was a better predictor of patient outcomes than RV%pred.

**Conclusion:**

This study demonstrates that both RV/TLC ratio and RV%pred are relevant indicators of hyperinflation in patients with severe emphysema in relation to patient-related outcomes. RV/TLC ratio is more strongly related to the patient-related outcomes than RV%pred.

**Electronic supplementary material:**

The online version of this article (10.1007/s00408-020-00368-9) contains supplementary material, which is available to authorized users.

## Introduction

Chronic obstructive pulmonary disease (COPD) is a progressive and incurable disease. This is initially reflected by a reduced forced expiratory volume in 1 s (FEV_1_) as well as a reduced FEV_1_/forced vital capacity (FVC) ratio [1, 2]. Over time, this will lead to a further decrease in expiratory flow and increase in lung static hyperinflation [3]. Patients will experience limitations in rest increasing with exercise as there is an increased end-expiratory lung volume. During exercise, the time for the lungs to empty is reduced, leading to incomplete lung emptying, which results in dynamic hyperinflation. Both static and dynamic hyperinflation are directly associated with patient-centered outcomes [4, 5]. The level of disability experienced among patients with COPD varies widely, but can be measured using commonly used tools such as the modified Medical Research Council (mMRC) dyspnea scale, the 6-min walk test (6MWT), and the St. George’s Respiratory Questionnaire (SGRQ) [6–9]. These outcomes can be improved by interventions that reduce static hyperinflation [3, 10] such as bronchodilators, breathing exercises, and surgical and bronchoscopic lung volume reduction (BLVR) techniques [11–13]. Especially for lung volume reduction modalities, the severity of static lung hyperinflation largely determines eligibility and success rate for these treatments [14, 15]. However, it is not exactly known what parameter should be used to optimally reflect hyperinflation. Therefore, the there is a need to find the best objective standards to identify patients who potentially benefit most from BLVR remains.

Various lung function parameters can be used to measure hyperinflation. The most commonly used parameters to reflect static hyperinflation are the residual volume (RV) and the ratio of RV to total lung capacity (RV/TLC). Another lung function parameters reflecting hyperinflation is the ratio of the inspiratory capacity (IC) to TLC, and a low IC/TLC ratio (< 0.25) has been demonstrated to be prognostically unfavorable [16]. Furthermore, forced vital capacity (FVC) can be easily measured with spirometry and is negatively affected by hyperinflation [17]. The purpose of this study was to define which of these parameters is the most optimal reflection of lung hyperinflation in emphysema patients in relation to patient-related outcomes.

## Methods

### Patients and Study Design

This was a retrospective cohort study that included patients who were participants in eight different previous studies (performed between October 2006 and April 2016, see online Tables A1 and A2 for details) evaluating a BLVR treatment [18–25]. All study patients gave written informed consent and all studies were approved by the local ethics committee.

### Pulmonary Function Testing

Pulmonary function was measured post bronchodilator (400 µg salbutamol) and according to ATS/ERS guidelines [26, 27]. The following variables were measured during spirometry: inspiratory vital capacity (IVC), FVC, and FEV_1_. Body plethysmography (Jaeger MasterScreen™ body plethysmograph (CareFusion, Germany)) was performed after the spirometry, and used to measure TLC, RV and functional residual capacity (FRC).

### Patient-Related Outcome Measurements

During the baseline visit the mMRC and SGRQ were obtained. The 6MWT was performed at baseline and in accordance with ATS guidelines, and percentages of predicted were calculated from normal values [6–9, 28].

### Outcome Measures

The primary outcome measures of interest were the correlation of RV/TLC ratio and RV as percentage of the predicted value (RV%pred) to the mMRC, SGRQ, and 6MWT. Other outcome measures were correlations of other potential measurements of hyperinflation: FVC%pred, IC/TLC ratio to the mMRC, SGRQ, and 6MWT.

### Statistical Analysis

Correlation coefficients were calculated to establish whether there was a correlation between pulmonary function variables and patient-related outcomes. When data were normally distributed, Pearson correlation was used. Spearman correlation was used when data were not normally distributed. Linear regression analysis was performed to evaluate the independent predictors of 6MWD and SGRQ and we included the primary outcome measures only. Variables with a univariate association with a p-value of < 0.20 were considered to be used in a linear regression model (method enter). The linear regression model was adjusted for age, gender, height, and weight. Highly correlating variables (correlation coefficient > 0.70) were not included in the model, because of multicollinearity. RV/TLC ratio, RV%pred, and FVC%pred were divided in categories and then used in the linear regression analyses as follows: RV/TLC < 50%, 50–55%, 55–58%, 58–62%, 62–65%, 65–70%, and > 70%; RV%pred < 175%, 175–200%, 200–225%, 225–250%, and > 250%; FVC%pred < 60%, 60–70%, 70–80%, 80–90%, 90–100%, and > 100%. A *p*-value of < 0.05 was considered statistically significant. IBM SPSS Statistics version 23 (IBM, NY, USA) was used for all analyses.

## Results

A total of 275 patients with severe COPD were included in this study, with one patient being excluded from further analysis because of missing body plethysmograph measurements. Thus, a total of 274 were used for the final analyses (See Table 1 for patient characteristics).

**Table 1:** Patient characteristics (*N* = 274)

Demographics	
Male/female	94/180
Age (years)	59 ± 8
Body Mass Index (kg/m^2^)	23.7 ± 3.6
Pack years (1/year)	37 ± 17
Pulmonary function	
FVC (Liter)	2.57 ± 0.83
FVC (% of predicted value)	76.07 ± 17.45
FEV_1_ (Liter)	0.74 ± 0.28
FEV_1_ (% of predicted value)	26.99 ± 8.59
FEV_1_/FVC (%)	29.39 ± 6.93
TLC (Liter)	7.84 ± 1.41
Predicted TLC (Liter)	5.77 ± 1.06
VC (Liter)	2.88 ± 0.87
FRC (Liter)	6.08 ± 1.23
RV (Liter)	4.94 ± 1.06
IC (Liter)	1.76 ± 0.59
Raw (kPa/Liter/second) (*N* = 245)	0.74 ± 0.28
SGaw, 1/(kPa*second) (*N* = 213)	0.26 ± 0.12
Ratio of RV to TLC (%)	63.12 ± 8.06
Ratio of RV to TLC predicted (%)	86.62 ± 16.05
Ratio of IC to TLC (%)	22.51 ± 6.41
Ratio of FVC to VC (%)	88.16 ± 8.23
Ratio of FRC to TLC (%)	77.49 ± 6.41
Patient related outcomes	
St. George's respiratory questionnaire, total score (*N* = 269)	61.2 ± 12.3
6-min walk distance (*N* = 273), meter	339 ± 95
% of predicted value	61 ± 17
Modified medical research council scale, grade (*N* = 265)	2.9 ± 0.7

Results are presented as mean ± standard deviation. Other parameters are presented as numbers

*FVC* forced vital capacity, *FEV*_*1*_ forced expiratory volume in 1 s, *TLC* total lung capacity, *VC* vital capacity, *FRC* forced residual capacity, *RV* residual volume, *IC* inspiratory capacity, *Raw* airway resistance, *SGaw* 1/airway resistance

**Fig. 1:**
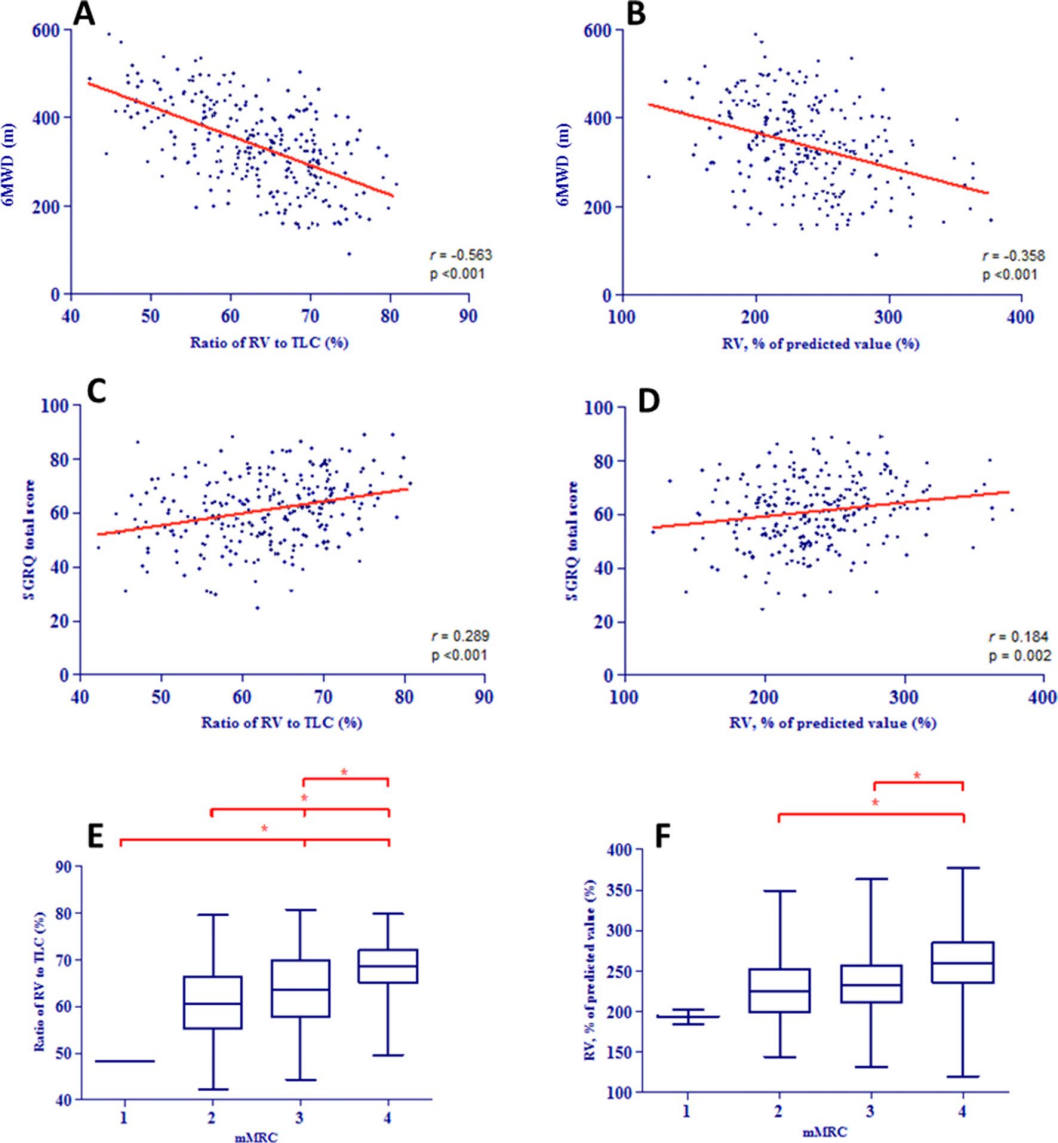
**a** scatterplot of 6-min walk test (6MWT) and residual volume/total lung capacity ratio (RV/TLC); **b** scatterplot of 6MWT and RV % of predicted; **c** scatterplot of the Saint George’s Respiratory Questionnaire (SGRQ) and RV/TLC ratio; **d** scatterplot of SGRQ and RV % of predicted; **e** boxplot of the modified medical Research Counsel dyspnea scale (mMRC) and RV/TLC ratio; **f** boxplot of mMRC and RV % of predicted. *Indicates *p* < 0.05

Significant correlations were found between both RV/TLC ratio and 6MWD (*r* =  − 0.563, *p* < 0.001) and between RV%pred and 6MWD (*r* =  − 0.358, *p* < 0.001). We found lower, but still statistically significant correlations of RV/TLC ratio and RV%pred with SGRQ (Fig. 1a–f) (See Table 2 for all correlations).

**Table 2:** Correlations overview

Variable	mMRC	*p*-value	SGRQ	*p*-value	6MWD	*p*-value
Pack years (years)	0.008	0.893	0.051	0.404	− 0.008	0.897
Age (years)	0.113*	0.067*	0.079*	0.198*	−** 0.141**	**0.019**
Height (centimeter)	− 0.103*	0.093*	− 0.090*	0.142*	**0.195**	**0.001**
Weight (kg)	− 0.020	0.742	− 0.030	0.623	0.047	0.436
Body Mass Index (kg/m^2^)	0.050	0.419	0.026	0.669	− 0.078*	0.200*
IVC (L)	** 0.307**	** < 0.001**	− **0.246**	** < 0.001**	**0.508**	** < 0.001**
IVC (% of predicted value)	−** 0.271**	** < 0.001**	−** 0.227**	** < 0.001**	**0.458**	** < 0.001**
VC (L)	−** 0.302**	** < 0.001**	−** 0.257**	** < 0.001**	**0.464**	** < 0.001**
FVC (L)	− **0.294**	** < 0.001**	−** 0.244**	** < 0.001**	**0.484**	** < 0.001**
FVC (% of predicted value)	−** 0.250**	** < 0.001**	−** 0.218**	** < 0.001**	**0.416**	** < 0.001**
FEV_1_ (L)	−** 0.328**	** < 0.001**	−** 0.199**	**0.001**	**0.542**	** < 0.001**
FEV_1_ (% of predicted value)	− **0.272**	** < 0.001**	−** 0.141**	**0.021**	**0.466**	** < 0.001**
TLC (L)	− 0.059	0.335	− 0.048	0.434	0.116*	0.055*
TLC (% of predicted value)	0.098*	0.110*	0.058	0.346	−** 0.127**	**0.037**
RV (L)	**0.169**	**0.006**	**0.134**	**0.028**	−** 0.254**	** < 0.001**
RV (% of predicted value)	**0.228**	** < 0.001**	**0.184**	**0.002**	−** 0.358**	** < 0.001**
FRC (L)	0.071	0.248	0.050	0.414	−** 0.129**	**0.033**
FRC (% of predicted value)	**0.206**	**0.001**	**0.145**	**0.017**	−** 0.335**	** < 0.001**
Raw (kPa/Liter/second)	**0.329**	** < 0.001**	**0.317**	** < 0.001**	−** 0.489**	** < 0.001**
Raw (% of predicted value)	**0.290**	** < 0.001**	**0.301**	** < 0.001**	−** 0.494**	** < 0.001**
SGaw, 1/(kPa*second)	− **0.273**	** < 0.001**	−** 0.236**	**0.001**	**0.512**	** < 0.001**
SGaw (% of predicted value)	− **0.289**	** < 0.001**	−** 0.260**	** < 0.001**	**0.553**	** < 0.001**
IC (L)	−** 0.302**	** < 0.001**	−** 0.227**	** < 0.001**	**0.529**	** < 0.001**
RV/TLC (%)	**0.354**	** < 0.001**	**0.289**	** < 0.001**	**0.563**	** < 0.001**
RV/TLCpredicted (%)	**0.298**	** < 0.001**	**0.228**	** < 0.001**	** 0.452**	** < 0.001**
Ratio of FVC to VC (%)	− 0.010	0.877	− 0.043	0.486	0.005	0.939
Ratio of IC to TLC (%)	−** 0.307**	** < 0.001**	−** 0.227**	** < 0.001**	**0.550**	** < 0.001**
Ratio of FRC to TLC (%)	**0.305**	** < 0.001**	**0.244**	** < 0.001**	− **0.547**	** < 0.001**
Ratio of FEV_1_ to FVC (%)	− **0.162**	**0.008**	− 0.004	0.953	**0.245**	** < 0.001**
SGRQ (points)	**0.518**	** < 0.001**	–	–	−** 0.368**	** < 0.001**
6MWD (meter)	−** 0.512**	** < 0.001**	− **0.368**	** < 0.001**	–	–
mMRC (points)	–	–	**0.518**	** < 0.001**	− **0.512**	** < 0.001**

*IVC* inspiratory vital capacity, *FVC* forced vital capacity, *FEV*_*1*_ forced expiratory volume in 1 s, *TLC* total lung capacity, *RV* residual volume, *FRC* forced residual capacity, *Raw* airway resistance, *SGaw* 1/airway resistance, *IC* inspiratory capacity, *mMRC* modified medical research council scale, *SGRQ* St. George's Respiratory Questionnaire, *6MWD* 6-min walk distance

Significant values (*p* < 0.05) were depicted in bold. * 0.05 ≥ *p* ≤ 0.20

Linear regression analyses showed that RV/TLC ratio, RV%pred, and FVC%pred are independent predictors of 6MWD (online supplement, Tables 3A–C). The model using RV/TLC ratio explained most of the variation in 6MWD (*R*^2^ = 0.476). When RV/TLC ratio increases by one percent, a patient walks approximately 4.8 m fewer (*b* =  − 4.812). Linear regression analyses of SGRQ showed that the primary outcome measures (RV/TLC ratio, RV%pred) and FVC%pred are not independent predictors of SGRQ (online supplement, Tables A3A–C). We also performed linear regression analyses with the primary outcome measures divided into categories. We found significant differences between the RV/TLC ratio categories. This model explained 47.3% of the variation in 6MWD (online supplement, Table A4A). Using this model, a hypothetical patient with a RV/TLC ratio of > 70% walked approximately 116 m less during a 6MWT compared to patients with a RV/TLC ratio < 50%. The model with RV%pred can be used to explain 41.4% of the variation in 6MWD outcome. When using RV%pred > 250% as reference value, all other categories were significantly different compared to this reference category. The model including FVC%pred (Table A4C) explained 46.7% of the variation in 6MWD outcome. In addition, linear regression analyses of SGRQ showed no significant differences between several categories of RV/TLC ratio, RV%pred, and FVC%pred (online supplement, Tables A5A–C).

## Discussion

The results of this study show that RV/TLC ratio and RV%pred both provide relevant information about the impact of hyperinflation on the patients quality of life and exercise tolerance, with RV/TLC ratio being the best predictor of the variation in outcomes.

Interestingly, the correlation of RV/TLC to patient-related outcomes is stronger than the correlation between RV%pred and patient-related outcomes. A possible explanation is that RV/TLC is calculated by dividing the actual measured RV in liters by the patients’ own actual measured TLC in liters, thus not being limited by predicted values based on length, age, and gender. In other words, RV%pred is a general parameter, with potential bias, making it a little less suitable for accurate individual measurement of hyperinflation. Our results are in line with an earlier study in surgical lung volume reduction patients, which reported a strong correlation between RV/TLC ratio and the reduction in FEV_1_ [29] (Table 3).

**Table 3:** Linear model of predictors of 6MWD (meter)

Variable	b	SE B	*β*	*p*-value
A				
RV/TLC	− 4.812	0.621	− 0.404	** < 0.001**
Ratio of FEV_1_ to FVC	2.584	0.666	0.187	** < 0.001**
Age	− 0.509	0.602	− 0.043	0.398
Gender	1.822	13.913	0.009	0.896
Height	1.925	0.792	0.174	**0.016**
Weight	− 1.219	0.424	− 0.159	**0.004**
mMRC	− 36.584	7.545	− 0.272	** < 0.001**
SGRQ	− 0.783	0.420	− 0.101	0.063
B				
RV_perc_pred	− 0.634	0.124	− 0.287	** < 0.001**
FEV_1_/ FVC	2.036	0.736	0.147	**0.006**
Age	− 2.242	0.638	− 0.189	**0.001**
Gender	− 4.087	14.715	− 0.020	0.781
Height	2.719	0.851	0.246	**0.002**
Weight	− 1.139	0.450	− 0.148	**0.012**
mMRC	− 42.776	7.898	− 0.318	** < 0.001**
SGRQ	− 0.925	0.445	− 0.119	**0.038**
C				
FVC_perc_pred	1.946	0.262	0.358	** < 0.001**
FEV_1_/ FVC	4.012	0.677	0.290	** < 0.001**
Age	− 1.913	0.595	− 0.161	**0.001**
Gender	− 22.788	13.836	− 0.113	0.101
Height	2.124	0.799	0.192	**0.008**
Weight	− 0.996	0.425	− 0.130	**0.020**
mMRC	− 35.882	7.636	− 0.266	** < 0.001**
SGRQ	− 1.009	0.420	− 0.130	**0.017**

(a) *R*^2^ =  0.476; (b) *R*^2^ = 0.413; (c)*R*^2^ = 0.467

*RV* residual volume, *TLC* total lung capacity, *RV_TLC_ratio* ratio of RV to TLC, *RV_perc_pred* residual volume, percentage of predicted value, *FVC_perc_pred* forced vital capacity, percentage of predicted value, *FEV*_*1*_ forced expiratory volume in 1 s, *FVC* forced vital capacity, *6MWD* 6-min walk distance, *mMRC* modified Medical Research Council dyspnea scale, *SGRQ* St. George’s Respiratory Questionnaire

Significant values (*p* < 0.05) were depicted in bold

The results of this study showed that the primary outcome variables (RV/TLC ratio, RV%pred) and FVC%pred correlated best with 6MWD and SGRQ. Many factors are known to contribute to the 6MWD in this patient group. Previous studies showed that 6MWD correlated to multiple pulmonary function variables such as RV/TLC ratio, FEV_1_, FEV_1_ as percentage of predicted value, FVC, and FEV_1_/FVC ratio, but also age, weight, gender, and height, and even SGRQ and mMRC explain a great variety in 6MWD [30, 31]. Even a learning curve could also be an influencing factor: the more often someone performs the test, the better this person understands in which manner the best result can be achieved [28, 32]. Contrary to the 6MWD, there are just minimal correlations between the pulmonary function test values and SGRQ. Furthermore, the independent predictors that were significant for 6MWD were not significant for the SGRQ. This is somewhat surprising, since the previous studies indicate a quite fair clinical significance of the SGRQ to other clinical outcomes [7, 8]. A possible explanation might be that this questionnaire is a more subjective measurement than the 6MWT. Furthermore, it may be that patients with the most advanced COPD (such as our now evaluated very severe emphysema patients) were not included in the initial validation studies of the SGRQ. This is also reflected by the fact that we recently showed that the minimal clinical important difference for the SGRQ total score for this patient group is a change of 7 points 6 months after intervention, whereas the validation literature showed a difference of only 4 points in a patient population with milder COPD [8, 33].

Looking at the mMRC, it might be concluded that this parameter was not as useful as we expected. Considering this is a categorical variable with a zero to four scale, it is perhaps less sensitive to detect feelings of breathlessness related to the level of hyperinflation. Another explanation for a low sensitivity might be that the mMRC is not a composite measure, but only measures the general dyspnea sensation. The results of this study are in line with an earlier study that demonstrated a discrepancy when comparing several scores from mMRC with COPD assessment test (CAT; health status) [34]. On the other hand, it can be noticed that in every linear regression model of the predictors of 6MWD and SGRQ, mMRC is a sensitive predictor. Therefore, the exact role of mMRC when determining hyperinflation remains unclear.

Next to RV/TLC ratio, FVC is also of great importance to display the health status after a procedure. A relative simple explanation can be that after lung volume reduction, the RV/TLC ratio improves and so does VC, and thus FVC. Previous research showed improved FVC is an important predictor of an improved FEV_1_ [35]. Also in our data, a high correlation was found between FVC and 6MWD (*r* = 0.484, *p* < 0.001), as well as significant, but slightly weaker correlations between FVC and SGRQ or mMRC. So FVC might be a relevant variable when trying to select patients who benefit most from BLVR. The potential advantage of using FVC as a “screening” tool is the fact that it can be derived from a simple spirometry (flow/volume measurement), making it a cheap and easy to use tool.

The FVC/VC ratio does not correlate significantly with any patient-related outcome variable (Table 2). This can be explained by the fact that both parameters are strongly associated, and thus leverage each other. The impact of FEV_1_/FVC ratio is not strongly correlated with patient-related outcomes as well. Generally, this ratio is used to indicate the amount of airflow limitation [36]. Also, this study showed a weak but significant correlation between this ratio and 6MWD (*r* =  − 0.245, *p* < 0.001). Furthermore, the correlation between FEV_1_/FVC ratio and SGRQ is not significant at all (*r* =  − 0.004, *p* = 0.953). A conclusion could be that FEV_1_/FVC ratio does not correspond to the clinical magnitude of hyperinflation and is therefore less suitable for the goal of this study.

Another variable we looked at was IC/TLC ratio. In our study population both functional residual capacity and TLC are increased. As a result, IC is decreased and a situation arises which is referred to as hyperinflation. IC/TLC ratio correlates significantly with all patient outcome variables, yet comparable with RV/TLC ratio. Therefore, it might be useful to keep this ratio in mind when trying to select the best patients for BLVR. The small downside of using IC/TLC, is that a separate IC maneuver needs to be performed to capture this data, while with measuring and calculating TLC, RV is readily available.

The goal of this study was to determine which of our selected parameters is the best reflection of hyperinflation in relation to patient-related outcomes. Much research has been conducted to discover the physiological concepts behind lung volume reduction, with patients having static hyperinflation, and reducing this by many different interventions being the main driver of response [37]. This study is one of the first to specify the best measure of static hyperinflation in this patient population

In conclusion, we showed that both RV/TLC ratio and RV%pred are relevant indicators of hyperinflation in relation to patient-related outcomes in patients with severe emphysema, with the RV/TLC ratio being the best predictor of the variation in baseline 6MWD. Further research is necessary to even more accurately determine other measures of hyperinflation that correlate with limitations experienced by patients.

## Electronic supplementary material

Below is the link to the electronic supplementary material.

Supplementary file1 (DOCX 39 kb)
