# Supplementary material for: Determining Static Hyperinflation in Patients with Severe Emphysema: Relation Between Lung Function Parameters and Patient-Related Outcomes
Source: Lung. 2020 Jun 28;198(4):629–36. doi: 10.1007/s00408-020-00368-9 (PMC7374464; doi:10.1007/s00408-020-00368-9)
Supplement: Supplementary file 1 — Supplementary file1 (DOCX 39 kb) [file 408_2020_368_MOESM1_ESM.docx]

**Table A1.** Study overview.^15-22^

| **Study** | **Reference** |
| --- | --- |
| I | *Shah et al.* Bronchoscopic lung-volume reduction with Exhale airway stents for emphysema (EASE trial): Randomised, sham-controlled, multicentre trial.^15^  Clinical Trial number: NCT00391612 |
| II | *Herth et al.* Radiological and clinical outcomes of using Chartis^TM^ to plan endobronchial valve treatment.^16^  Clinical Trial number: NCT01101958 |
| III | *Klooster et al.* Endobronchial Valves for Emphysema without Interlobar Collateral Ventilation.^17^  Clinical Trial number: NTR2876 |
| IV | *Valipour et al.* Endobronchial valve therapy in patients with homogeneous emphysema: results from the IMPACT study.^18^  Clinical Trial number: NCT02025205 |
| V | *Slebos et al.* Bronchoscopic lung volume reduction coil treatment of patients with severe heterogeneous emphysema.^19^  Clinical Trial number: NCT01220908 |
| VI | *Deslee et al.* Lung volume reduction coil treatment for patients with severe emphysema: a European multicentre trial.^20^  Clinical Trial number: NCT01328899 |
| VII | *Klooster et al.* Lung volume reduction coil treatment in chronic obstructive pulmonary disease patients with homogeneous emphysema: a prospective feasibility trial.^21^  Clinical Trial number: NCT01421082 |
| VIII | *Sciurba et al.* Effect of Endobronchial Coils vs Usual Care on Exercise Tolerance in Patients With Severe Emphysema: The RENEW Randomized Clinical Trial.^22^  Clinical Trial number: NCT01608490 |

**Table A2.** Combined global inclusion and exclusion criteria.^15-22^

| ***Inclusion criteria*** |
| --- |
| Age >35 years |
| Non-smokers for a period of 8 weeks or 6 months |
| FEV_1_ <50% of predicted |
| RV >175% of predicted (body plethysmography) |
| RV/TLC ≥0.65 (body plethysmography) |
| TLC >100% of predicted |
| mMRC ≥2 |
| Diagnosed with severe emphysematous type of COPD |
|  |
| ***Exclusion criteria*** |
| Change in FEV_1_ >20% post-bronchodilator |
| Inability to walk >140m in 6 min |
| Pulmonary hypertension defined by right ventricular pressure >50 mmHg |
| Evidence of active pulmonary infection |
| History of more than 3 exacerbations with hospitalizations over the past 12 months |
| Clinically significant bronchiectasis |
| Diagnosed with alpha-1 antitrypsin deficiency^*^ |
| DLCO <20% predicted |
| Previous LVRS, BLVR, lung transplantation, lobectomy or other devices to treat COPD in either lung |
| Giant bullae: more than one-third lung volume |
| >20 mg prednisone (or equivalent) daily |
| Antiplatelet agent (e.g. Plavix) which cannot be stopped prior to procedure |

^*^AATD was mentioned only as an exclusion criteria in IV^18^ and VIII^22^

**Table A3A.** Linear model of predictors of SGRQ (points).

| **Variable** | **b** | **SE B** | ***β*** | **p-value** |
| --- | --- | --- | --- | --- |
| RV/TLC | 0.138 | 0.102 | .090 | 0.175 |
| FEV_1_/FVC | 0.288 | 0.100 | .161 | **0.004** |
| Age | -0.062 | 0.089 | -.041 | 0.484 |
| Gender | -2.344 | 2.052 | -.090 | 0.254 |
| Height | -0.034 | 0.118 | -.024 | 0.774 |
| Weight | -0.028 | 0.064 | -.028 | 0.661 |
| mMRC | 7.863 | 1.057 | .453 | **<0.001** |
| 6MWD | -0.017 | 0.009 | -.133 | 0.063 |

Note. R^2^ = .312

RV = residual volume**;** TLC = total lung capacity; RV_TLC_ratio = ratio of RV to TLC; FEV_1_ = forced expiratory volume in 1 second; FVC = forced vital capacity; 6MWD = 6-minute walk distance; mMRC = modified Medical Research Council dyspnea scale; SGRQ = St. George’s Respiratory Questionnaire. Significant values (p<0.05) were depicted in bold.

**Table A3B.** Linear model of predictors of SGRQ (points).

| **Variable** | **b** | **SE B** | ***β*** | **p-value** |
| --- | --- | --- | --- | --- |
| RV_perc_pred | 0.031 | 0.018 | .108 | 0.088 |
| FEV_1_/FVC | 0.328 | 0.102 | .184 | **0.001** |
| Age | 0.001 | 0.091 | .000 | 0.995 |
| Gender | -2.436 | 2.046 | -.094 | 0.235 |
| Height | -0.070 | 0.121 | -.049 | 0.563 |
| Weight | -0.024 | 0.063 | -.025 | 0.702 |
| mMRC | 7.886 | 1.053 | .453 | **<0.001** |
| 6MWD | -0.018 | 0.009 | -.139 | **0.038** |

Note. R^2^ = .314.

RV = residual volume. RV_perc_pred = residual volume, percentage of predicted value; FEV_1_ = forced expiratory volume in 1 second; FVC = forced vital capacity; 6MWD = 6-minute walk distance; mMRC = modified Medical Research Council dyspnea scale; SGRQ = St. George’s Respiratory Questionnaire. Significant values (p<0.05) were depicted in bold.

**Table A3C.** Linear model of predictors of SGRQ (points).

| **Variable** | **b** | **SE B** | ***β*** | **p-value** |
| --- | --- | --- | --- | --- |
| FVC_perc_pred | -0.010 | 0.043 | -.014 | 0.820 |
| FEV_1_/FVC | 0.283 | 0.105 | .159 | **0.007** |
| Age | -0.039 | 0.089 | -.025 | 0.664 |
| Gender | -1.867 | 2.044 | -.072 | 0.362 |
| Height | -0.027 | 0.119 | -.019 | 0.820 |
| Weight | -0.043 | 0.063 | -.043 | 0.498 |
| mMRC | 7.960 | 1.060 | 0.458 | **<0.001** |
| 6MWD | -0.022 | 0.009 | -.170 | **0.017** |

Note. R^2^ = .307.

FVC_perc_pred = forced vital capacity, percentage of predicted value; FEV_1_ = forced expiratory volume in 1 second; FVC = forced vital capacity; 6MWD = 6-minute walk distance; mMRC = modified Medical Research Council dyspnea scale; SGRQ = St. George’s Respiratory Questionnaire. Significant values (p<0.05) were depicted in bold.

**Table A4A.** Linear model of predictors of 6MWD (meter).

| **Variable** | **b** | **SE B** | ***β*** | **p-value** |
| --- | --- | --- | --- | --- |
| RV/TLC <50% | 116.294 | 21.177 | .313 | **<0.001** |
| RV/TLC 50-55% | 98.508 | 18.138 | .295 | **<0.001** |
| RV/TLC 55-58% | 74.288 | 17.269 | .242 | **<0.001** |
| RV/TLC 58-62% | 57.601 | 15.846 | .206 | **<0.001** |
| RV/TLC 62-65% | 37.953 | 15.604 | .134 | **0.016** |
| RV/TLC 65-70% | 11.984 | 13.108 | .053 | 0.361 |
| RV/TLC >70% |  |  |  |  |
| FEV_1_/FVC | 2.536 | 0.699 | .183 | **<0.001** |
| Age | -0.409 | 0.624 | -.034 | 0.513 |
| Gender | 3.259 | 14.410 | .016 | 0.821 |
| Height | 2.084 | 0.821 | .189 | **0.012** |
| Weight | -1.234 | 0.431 | -.161 | **0.005** |
| mMRC | -37.142 | 7.687 | -.276 | **<0.001** |
| SGRQ | -0.852 | 0.425 | -.110 | **0.046** |

Note. R^2^ = .473.

RV = residual volume**;** TLC = total lung capacity; RV_TLC_ratio = ratio of RV to TLC; FEV_1_ = forced expiratory volume in 1 second; FVC = forced vital capacity; 6MWD = 6-minute walk distance; mMRC = modified Medical Research Council dyspnea scale; SGRQ = St. George’s Respiratory Questionnaire. Significant values (p<0.05) were depicted in bold.

**Table A4B.** Linear model of predictors of 6MWD (meter).

| **Variable** | **b** | **SE B** | ***β*** | **p-value** |
| --- | --- | --- | --- | --- |
| RV_perc_pred_<175 | 76.068 | 21.435 | .195 | **<0.001** |
| RV_perc_pred_175-200 | 57.791 | 16.494 | .194 | **0.001** |
| RV_perc_pred_200-225 | 51.629 | 13.980 | .217 | **<0.001** |
| RV_perc_pred_225-250 | 47.629 | 12.068 | .223 | **<0.001** |
| RV_perc_pred_>250 |  |  |  |  |
| FEV_1_/FVC | 2.466 | 0.737 | .178 | **0.001** |
| Age | -2.062 | 0.636 | -.174 | **0.001** |
| Gender | -6.625 | 14.811 | -.033 | 0.655 |
| Height | 2.562 | 0.852 | .232 | **0.003** |
| Weight | -1.016 | 0.450 | -.132 | **0.025** |
| mMRC | -42.064 | 8.070 | -.312 | **<0.001** |
| SGRQ | -0.997 | 0.446 | -.129 | **0.026** |

Note. R^2^ = .414.

RV = residual volume. RV_perc_pred = residual volume, percentage of predicted value; FEV_1_ = forced expiratory volume in 1 second; FVC = forced vital capacity; 6MWD = 6-minute walk distance; mMRC = modified Medical Research Council dyspnea scale; SGRQ = St. George’s Respiratory Questionnaire. Significant values (p<0.05) were depicted in bold.

**Table A4C.** Linear model of predictors of 6MWD (meter).

| **Variable** | **b** | **SE B** | ***β*** | **p-value** |
| --- | --- | --- | --- | --- |
| FVC_perc_pred_>100 | 97.173 | 19.099 | .280 | **<0.001** |
| FVC_perc_pred_90-100 | 74.472 | 16.718 | .260 | **<0.001** |
| FVC_perc_pred_80-90 | 48.461 | 16.057 | .181 | **0.003** |
| FVC_perc_pred_70-80 | 16.072 | 14.015 | .071 | 0.253 |
| FVC_perc_pred_60-70 | -4.128 | 14.301 | -.018 | 0.773 |
| FVC_perc_pred_<60 |  |  |  |  |
| FEV_1_/FVC | 3.790 | 0.688 | .274 | **<0.001** |
| Age | -1.626 | 0.601 | -.137 | **0.007** |
| Gender | -21.808 | 14.095 | -.108 | 0.123 |
| Height | 2.183 | 0.813 | .198 | **0.008** |
| Weight | -1.130 | 0.431 | -.147 | **0.009** |
| mMRC | -35.870 | 7.776 | -.266 | **<0.001** |
| SGRQ | -1.124 | 0.427 | -.145 | **0.009** |

Note. R^2^ = .467.

FVC_perc_pred = forced vital capacity, percentage of predicted value; FEV_1_ = forced expiratory volume in 1 second; FVC = forced vital capacity; 6MWD = 6-minute walk distance; mMRC = modified Medical Research Council dyspnea scale; SGRQ = St. George’s Respiratory Questionnaire. Significant values (p<0.05) were depicted in bold.

**Table A5A.** Linear model of predictors of SGRQ (points).

| **Variable** | **b** | **SE B** | ***β*** | **p-value** |
| --- | --- | --- | --- | --- |
| RV/TLC <50% | -3.069 | 3.295 | -.064 | 0.353 |
| RV/TLC 50-55% | -3.194 | 2.817 | -.074 | 0.258 |
| RV/TLC 55-58% | -1.975 | 2.633 | -.050 | 0.454 |
| RV/TLC 58-62% | -3.044 | 2.387 | -.084 | 0.203 |
| RV/TLC 62-65% | -2.102 | 2.321 | -.058 | 0.366 |
| RV/TLC 65-70% | -2.169 | 1.929 | -.075 | 0.262 |
| RV/TLC >70% |  |  |  |  |
| FEV_1_/FVC | 0.283 | 0.104 | .159 | **0.007** |
| Age | -0.048 | 0.092 | -.032 | 0.599 |
| Gender | -2.058 | 2.119 | -.079 | 0.332 |
| Height | -0.035 | 0.122 | -.025 | 0.774 |
| Weight | -0.030 | 0.065 | -.030 | 0.646 |
| mMRC | 7.795 | 1.077 | .449 | **<0.001** |
| 6MWD | -0.018 | 0.009 | -.143 | **0.046** |

Note. R^2^ = .313.

RV = residual volume**;** TLC = total lung capacity; RV_TLC_ratio = ratio of RV to TLC; FEV_1_ = forced expiratory volume in 1 second; FVC = forced vital capacity; 6MWD = 6-minute walk distance; mMRC = modified Medical Research Council dyspnea scale; SGRQ = St. George’s Respiratory Questionnaire. Significant values (p<0.05) were depicted in bold.

**Table A5B.** Linear model of predictors of SGRQ (points).

| **Variable** | **b** | **SE B** | ***β*** | **p-value** |
| --- | --- | --- | --- | --- |
| RV_perc_pred_<175 | -3.207 | 3.058 | -.064 | 0.295 |
| RV_perc_pred_175-200 | -1.424 | 2.355 | -.037 | 0.546 |
| RV_perc_pred_200-225 | -0.121 | 2.003 | -.004 | 0.952 |
| RV_perc_pred_225-250 | -2.339 | 1.729 | -.085 | 0.177 |
| RV_perc_pred_>250 |  |  |  |  |
| FEV_1_/FVC | 0.284 | 0.104 | .159 | **0.007** |
| Age | -0.023 | 0.091 | -.015 | 0.796 |
| Gender | -2.026 | 2.064 | -.078 | 0.327 |
| Height | -0.048 | 0.121 | -.034 | 0.691 |
| Weight | -0.036 | 0.063 | -.037 | 0.569 |
| mMRC | 8.066 | 1.071 | .465 | **<0.001** |
| 6MWD | -0.019 | 0.009 | -.150 | **0.026** |

Note. R^2^ = .315.

RV = residual volume. RV_perc_pred = residual volume, percentage of predicted value; FEV_1_ = forced expiratory volume in 1 second; FVC = forced vital capacity; 6MWD = 6-minute walk distance; mMRC = modified Medical Research Council dyspnea scale; SGRQ = St. George’s Respiratory Questionnaire. Significant values (p<0.05) were depicted in bold.

**Table A5C.** Linear model of predictors of SGRQ (points).

| **Variable** | **b** | **SE B** | ***β*** | **p-value** |
| --- | --- | --- | --- | --- |
| FVC_perc_pred_>100 | -2.704 | 2.917 | -.060 | 0.355 |
| FVC_perc_pred_90-100 | -0.291 | 2.529 | -.008 | 0.909 |
| FVC_perc_pred_80-90 | -1.270 | 2.380 | -.037 | 0.594 |
| FVC_perc_pred_70-80 | -3.712 | 2.033 | -.127 | 0.069 |
| FVC_perc_pred_60-70 | -2.841 | 2.076 | -.095 | 0.172 |
| FVC_perc_pred_<60 |  |  |  |  |
| FEV_1_/FVC | 0.278 | 0.105 | .156 | **0.008** |
| Age | -0.029 | 0.089 | -.019 | 0.745 |
| Gender | -1.816 | 2.060 | -.070 | 0.379 |
| Height | -0.016 | 0.120 | -.011 | 0.892 |
| Weight | -0.051 | 0.064 | -.052 | 0.421 |
| mMRC | 7.896 | 1.070 | 0.455 | **<0.001** |
| 6MWD | -0.024 | 0.009 | -.185 | **0.009** |

Note. R^2^ = .320.

FVC_perc_pred = forced vital capacity, percentage of predicted value; FEV_1_ = forced expiratory volume in 1 second; FVC = forced vital capacity; 6MWD = 6-minute walk distance; mMRC = modified Medical Research Council dyspnea scale; SGRQ = St. George’s Respiratory Questionnaire. Significant values (p<0.05) were depicted in bold.
